# Workplace Violence Prevention in Forensic Psychiatric Nursing: A Five‐Year Qualitative Content Analysis of Incident Reports From a Finnish Forensic Psychiatric Hospital

**DOI:** 10.1111/jpm.70152

**Published:** 2026-05-22

**Authors:** Matias Karvonen, Riitta Askola, Emilia Laukkanen, Anssi Kuosmanen, Lauri Kuosmanen

**Affiliations:** ^1^ Department of Nursing Science University of Eastern Finland Kuopio Finland; ^2^ Health and Social Care Education Unit South‐Eastern Finland University of Applied Sciences Mikkeli Finland; ^3^ Department of Nursing Science University of Turku Turku Finland; ^4^ Savonia University of Applied Sciences Kuopio Finland; ^5^ Niuvaniemi Hospital Kuopio Finland

**Keywords:** forensic psychiatry, incident reporting system, psychiatric hospital, psychiatric nursing, workplace violence

## Abstract

**Introduction:**

Forensic psychiatric nursing staff are exposed to workplace violence, which harms well‐being and may undermine safe care.

**Aim/Question:**

To identify preventive factors for workplace violence on forensic psychiatric wards as reported in incident reports by nursing staff and nurse managers.

**Method:**

Qualitative inductive content analysis of workplace violence incident reports over 5 years at one Finnish forensic psychiatric hospital, focusing on prevention‐oriented free‐text fields. Categories were descriptively quantified as the number (%) of reports containing each category.

**Results:**

Incident reports portrayed workplace violence prevention as a layered entity covering clinical stabilisation, staffing and work organisation, anticipatory routines, therapeutic interaction and de‐escalation, environmental and technological safety measures, restrictions and external‐risk control, and information flow, debriefing and organisational learning.

**Discussion:**

Clinical, organisational and environmental factors were intertwined in prevention. Incident report narratives and measures may provide a qualitative source for examining prevention‐related practices and follow‐up actions in forensic psychiatric care.

**Limitations:**

Single‐hospital data, possible underreporting and a nursing‐only focus limit transferability.

**Implications for Practice:**

Following recommendations emerging from incident reports, such as ensuring adequate staffing, providing training and maintaining safe work environments, may support workplace violence prevention.

**Recommendations:**

Forensic psychiatric inpatient services may benefit from strengthening layered prevention strategies and from using incident report narratives to inform local policy and practice.

**Relevance Statement:**

Workplace violence is a persistent occupational hazard in forensic psychiatric nursing, yet little is known about how prevention is described in workplace violence incident reports. This study analyses prevention‐oriented incident report entries from a Finnish forensic psychiatric hospital to examine how nursing staff and nurse managers describe workplace violence prevention. Prevention was described as a layered entity involving clinical stabilisation, staffing and competence, anticipatory practices, therapeutic interaction, environmental and safety measures, restrictions and external‐risk control, and post‐incident information flow, debriefing and follow‐up actions. These findings may inform use of incident reports and prevention planning in similar secure forensic inpatient settings.

## Accessible Summary

- What is known on the subject
  ○ Forensic psychiatric wards care for people with severe mental health conditions and high‐risk patients; violence towards staff is frequent and harmful.
  ○ Many strategies to prevent violence have been proposed; however, these are based on mixed evidence. Little is known about what preventive actions and conditions are documented in incident reports from everyday forensic ward practice.
- Originality
  ○ This study analysed 5 years of workplace violence incident reports from a Finnish forensic psychiatric hospital and focused on what nursing staff and nurse managers documented about preventing such events.
  ○ It shows that workplace violence prevention is layered and involves a combination of various factors, that is, patients' mental stability, having enough skilled staff, risk anticipation, clear routines and limits, a safe environment and security technology, patient restrictions and structured information sharing, reporting and debriefing.
- Significance
  ○ The findings support investing in adequate and experienced staffing, proactive clinical stabilisation through timely medication review and adjustment, supporting regular sleep and daily rhythms, and reducing overload, alongside safe ward design and security technologies and clear, consistently applied rules and procedures.
  ○ Services may use information documented in incident reporting systems not only to count incidents but also to support local reflection, learning and policy development.
  ○ Better prevention of workplace violence may support patient safety and recovery‐oriented care and may help protect the well‐being and retention of forensic mental health staff.

## 1 Introduction

Working in forensic psychiatric care settings exposes nurses to the risk of workplace violence (Ireland et al. 2021; Newman et al. 2024). According to Kelly et al. (2015) and Newman et al. (2021), the most common forms of workplace violence (WPV) are verbal, physical and sexual violence. As many as 30%–40% of forensic psychiatric patients engage in violent behaviour during a 12‐month period of hospitalisation (Broderick et al. 2015) and most (approximately 70%) patient‐perpetrated violent incidents in forensic psychiatry are directed at other patients rather than staff (Bader et al. 2014). Nevertheless, a substantial proportion (70%–100%) of forensic psychiatric nursing staff report having experienced WPV in the past 12 months (Kelly et al. 2015; Niu et al. 2019; Newman et al. 2021, 2024). It should be noted that a small group of patients accounts for a large share of violent incidents on forensic psychiatric wards (Broderick et al. 2015).

WPV encountered by forensic psychiatric nurses is associated with poorer physical and mental health (van Leeuwen and Harte 2017; Newman et al. 2024; Bloemendaal et al. 2024), post‐traumatic stress disorder (Ireland et al. 2021) and increased stress and burnout (Kobayashi et al. 2020; Ireland et al. 2021). Experiencing WPV also leads to moral injury (Morris et al. 2022) and to more sickness absence among nurses (Newman et al. 2024). From a patient perspective, WPV may prolong the treatment episodes of forensic psychiatric patients and undermine the therapeutic climate on the ward (Verstegen et al. 2017; Aerts et al. 2023).

Managing violence on forensic psychiatric wards requires clear organisational policies and procedures and structured violence‐management training. Post‐incident debriefings, multidisciplinary collaboration, a supportive leadership culture and proactive risk assessments can help reduce WPV (O'Sullivan et al. 2020; Urheim et al. 2020; Asikainen et al. 2023). However, the evidence regarding de‐escalation training and its impact on reducing WPV in forensic psychiatric wards is mixed (Johnston et al. 2022; Brenig et al. 2023). The systematic risk assessment of WPV (e.g., based on DASA) and structured incident recording are associated with WPV prevention through improved anticipation, learning and decision‐making (Cowman et al. 2017; Ogonah et al. 2023).

In addition, the routine reporting of WPV is essential for detecting risk factors and working towards prevention. For the purposes of this study, we analysed records exported from the hospital's incident reporting system, which is widely used across Finnish health and social care facilities. A hospital's incident‐reporting system data support patient and staff safety by highlighting hazards and vulnerable points within care pathways (Hyvämäki et al. 2023). The system is intended to document safety‐related events, including incidents that expose employees or patients to danger such as threats, assaults, patient‐to‐patient violence and self‐harm attempts, regardless of whether any actual harm occurred, and it also records near‐misses (Awanic 2020).

Recent reviews in psychiatric and other healthcare settings summarise a wide range of WPV prevention strategies such as staff training, structured risk assessments, environmental modifications and multicomponent organisational programmes; however, they consistently report low‐quality evidence and only modest or uncertain effects, especially in inpatient and forensic psychiatry (Brenig et al. 2023; O'Brien et al. 2024). Qualitative studies underline the important role of everyday ward practices, staffing arrangements and ward climate in preventing aggression, yet these investigations are lacking in number and rarely focused on forensic wards (Lantta et al. 2016; Brunero et al. 2024). Emerging work on patient safety and incident reporting systems shows that staff‐reported violence events constitute a rich but underused resource for guiding targeted WPV prevention; although the preventive actions described in these reports have seldom been examined systematically (Kuosmanen et al. 2022; Clercx et al. 2025). This leaves a knowledge gap with regard to how forensic nursing staff and nurse managers conceptualise and operationalise preventive factors in their daily work, especially when viewed through staff‐reported WPV incident data. To translate WPV incidents into effective prevention, prevention‐oriented qualitative entries in incident reports, including narrative descriptions and documentation of implemented measures, are needed to reveal modifiable antecedents and concrete actions that incident counts alone cannot capture.

## 2 Aims

The aim of this study was to identify preventive factors for WPV on forensic psychiatric wards as reported in nursing staff and nurse managers' entries in incident reports.

The following research questions were addressed:

1. Which preventive factors are documented in nursing staff entries in incident reports for preventing WPV on forensic psychiatric wards?
2. Which preventive factors are documented in nurse managers' entries in incident reports for preventing WPV on forensic psychiatric wards?

## 3 Materials and Methods

### 3.1 Design and Settings

This study employed a qualitative descriptive design, using inductive content analysis of staff‐reported workplace violence (WPV) incident reports submitted to a Finnish forensic psychiatric hospital over a 5‐year period. The hospital provides highly specialised inpatient forensic psychiatric care and conducts forensic psychiatric mental state examinations. It treats patients who are considered dangerous and/or particularly difficult to treat, who account for almost 40% of those in forensic psychiatric care in Finland (Seppänen et al. 2020; Kuosmanen et al. 2022). The hospital under study comprises 297 patient beds across 13 adult forensic psychiatric wards and one specialised ward for underage patients, and it provides approximately 102,400 inpatient days per year on average.

This study used records from the web‐based incident reporting system routinely completed by staff at a forensic psychiatric hospital in Finland. WPV incidents can be reported by any member of the hospital's healthcare staff. Staff are instructed to submit an incident report whenever a situation involving WPV, threat of violence, or a near‐miss event occurs. Reporting practices are based on institutional guidelines that specify when an incident report should be submitted and what information regarding the event, persons involved, and contributing factors should be documented.

Approximately 550 staff members work at the hospital, of whom around 340 belong to the nursing staff. The nursing staff includes practical nurses and registered nurses working in psychiatric care roles. Almost all nursing staff have completed basic violence management training.

### 3.2 Data Collection

At the hospital under study, we extracted all eligible WPV incident reports recorded in the organisation's electronic incident reporting system between 1 January 2020 and 31 December 2024, constituting a consecutive census of eligible reports during the study period. For the purposes of this study, eligible reports were those filed by nursing staff, classified as violence events, and documenting patient‐perpetrated aggression towards staff on forensic inpatient wards. In total, 1018 WPV incident reports were identified, of which 956 were included in the analysis. Duplicates and records with critical missing information were excluded according to prespecified rules.

WPV is recorded as an occupational safety incident report and can be submitted by staff from all professional groups. Each event is classified as a near miss, a situation that could have resulted in harm, or an incident involving injury to staff. Reports include structured fields (e.g., unit, time and location, reporter's professional group, hazard type), and in all included reports the hazard type was recorded as violence. Structured incident descriptors (e.g., unit, time and location) were not analysed, as the study focused on WPV prevention‐oriented narrative fields and the implemented measures section.

Reporters provide a free‐text account of the event (e.g., the task underway when the situation began, how the incident unfolded, who was involved, and perceived contributing factors). Following submission, the nurse manager reviews the report and completes the implemented measures section, documenting follow‐up actions intended to prevent recurrence. The incident reporting system is intended to support collective reflection, improvement, and learning within the work community (Awanic 2020).

### 3.3 Ethical Considerations

The reporting dataset contained no patient names and no direct personal identifiers. The dataset contained occasional staff names, which were removed prior to analysis. After this, the researchers had access only to anonymised, exported incident report data. The dataset then contained no direct identifiers, and the analysis focused on narrative text content rather than individual cases. A privacy notice and a data protection impact assessment were prepared for the study. Under Finnish legislation, a separate ethical review was not required (Finnish National Board on Research Integrity, TENK 2019). The data were obtained after applying for a research permit from the hospital, which was granted by the hospital's chief physician. The security manager provided the data to the researcher.

### 3.4 Data Analysis

**FIGURE 1:**
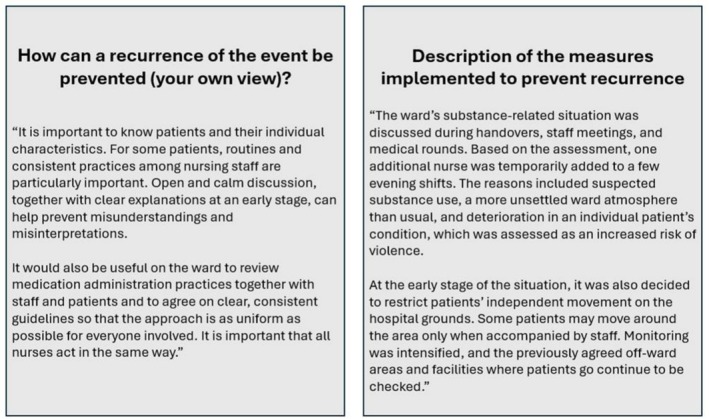
Example of WPV prevention‐oriented free‐text fields in the incident reporting system (composite and anonymized).

Among the included WPV incident reports (*n* = 956), nursing staff completed the field ‘How can a recurrence of the event be prevented (your own view)?’ in 552 cases. After removing duplicates and erroneous entries, 509 reports remained for analysis. Similarly, nurse managers completed the field ‘Description of the measures implemented to prevent recurrence’ in 539 reports. After removing duplicates and erroneous entries, 524 reports were included in the analysis. To illustrate the nature of the analysed free‐text material, Figure 1 presents a composite exemplar of the two WPV prevention‐oriented fields included in this study.

The dataset consisted of free‐text responses from nursing staff (*n* = 509) and nurse managers (*n* = 524) regarding the prevention of WPV incidents. The analysis was limited to incident reports filed by nursing staff to ensure that the material reflected WPV prevention‐related content documented in nursing staff entries and the corresponding nurse manager follow‐up within the same reports. In forensic psychiatric inpatient care, nursing staff represent the professional group most consistently exposed to direct patient contact and WPV situations. Consistent with this, Kuosmanen et al. (2022) reported that among 2521 incidents in a Finnish forensic psychiatric hospital, 64% were reported by registered nurses and 29% by practical nurses.

The analysis was carried out using inductive content analysis (Elo and Kyngäs 2008; Elo et al. 2014). The analysis was conducted by the first author. To enhance analytic rigour, coding decisions, category development and interpretations were regularly discussed within the research team. One member of the research team was employed by the study hospital, which supported contextual understanding of the data. Inductive content analysis is recommended when previous knowledge of a phenomenon is limited or fragmented and the aim is to allow categories to emerge from the data rather than to test a pre‐existing framework (Elo and Kyngäs 2008; Elo et al. 2014). To enhance trustworthiness, we followed the guidelines for qualitative content analysis described by Elo et al. (2014) including iterative reading of the data and careful documentation of analytic decisions. This approach was appropriate in our study because there is no established model governing how forensic nursing staff and nurse managers describe preventive factors in WPV incident reports.

The material included in the analysis comprised a total of 112 pages of text (58 pages of nurse manager entries and 54 pages of nursing staff entries), formatted in 12‐point Times New Roman with 1.5 line spacing. The data were imported into ATLAS.ti for analysis. The unit of analysis was a meaning unit (a sentence or part of a sentence) containing a single prevention‐related idea documented in nursing staff or nurse manager incident report entries. This unit size was chosen because preventive ideas were typically expressed as discrete, sentence‐level statements.

Using open coding, meaning units were labelled with descriptive codes that stayed close to the source text. Meaning units addressing the research questions were condensed at the sentence‐level from the free‐text sections of incident reports (*n* = 509 nursing staff entries; *n* = 524 nurse managers' entries) and then compared and grouped into higher‐order categories (*n* = 25 for nursing staff; *n* = 31 for nurse managers). Through constant comparison, these were further abstracted into main categories (*n* = 10; *n* = 13) and overarching categories (*n* = 5; *n* = 7), respectively. The resulting categories were named using concepts that describe their content (Polit and Beck 2017).

Table 1 provides an illustrative example of the stepwise abstraction process using one higher‐order category, whereas the full category structures for nursing staff and nurse managers are presented in Tables 2 and 3.

**TABLE 1:** Example illustrating the progression of inductive content analysis for the higher‐order category ‘Nurse staffing levels’.

Original expression	Condensed meaning unit	Open code	Subcategory	Higher‐order category
‘A sufficient number of nursing staff on the evening shift’	Ensure adequate staffing on evening shifts	Adequate evening shift staffing	Adequate staffing across shifts	Nurse staffing levels
‘A sufficient number of staff on each shift’	Maintain sufficient staffing across shifts	Adequate staffing on each shift		
‘Adequate staffing on site and calling for help in time’	Ensure staffing is adequate and summon support early	Early backup and timely assistance	Early backup and timely assistance	
‘More staff present in similar situations’	Increase staff presence in high‐risk situations	Need for additional staff in high‐risk situations	Staff presence in high‐risk situations	
‘There must be enough nurses present in the seclusion room and when caring for a secluded patient’	Ensure adequate staffing for seclusion care	Staffing for seclusion care	Staffing in seclusion care	
‘The nurses' heavy workload and low numbers fray the patients' nerves when their needs cannot be met on time’	Add staff to meet patient needs promptly and reduce escalation	Workload‐related delays increase risk	Workload‐related delays in meeting patient needs	

**TABLE 2:** Higher‐order categories of workplace violence prevention reported by nursing staff in incident reports (*n* = 509).

Overarching category	Main category	Higher‐order category	Reports, *n* (%)
Stabilising the patient's condition and reducing stressors	Clinical stabilisation	Optimising medication	50 (9.8)
		Adjusting ECT treatment	23 (4.5)
		Sleep, rest and daily rhythm	24 (4.7)
		Psychoeducation and guidance	13 (2.6)
	Management of patient stressors	Reducing stimuli and overload for patients	13 (2.6)
Ensuring a safe work environment and adequate nurse staffing	Resources and expertise	Nurse staffing levels	77 (15.1)
		Nursing staff work experience	30 (5.9)
		Adequate number of male nurses	19 (3.7)
		Multidisciplinary discussion/consultation	17 (3.3)
		Orientation and safety training	4 (0.8)
	Work organisation and patient placement	Ward transfers and unit structure	14 (2.8)
		Work allocation and one‐to‐one observation arrangements	24 (4.7)
		Resource‐ and system‐level constraints	40 (7.9)
Anticipation, consistency and limit‐setting with patients	Anticipation and consistent practices	Recognition of warning signs and early intervention	72 (14.1)
		Consistent policies and procedures	29 (5.7)
		Operational plans for high‐risk situations	5 (1.0)
	Limit‐setting and supervision	Control of substances and restrictions on incoming parcels	26 (5.1)
		Restrictions on phone use and external contacts	15 (2.9)
		Smoking management	10 (2.0)
Environment and safety technology	Environment and safety technology	Doors, locks and alarms	6 (1.2)
		Protective equipment and safety aids	6 (1.2)
		Restraints and restraint practices	15 (2.9)
		Control of hazardous objects and safe medication dispensing	18 (3.5)
	Seclusion measures	Seclusion and functionality of seclusion facilities	36 (7.1)
Therapeutic engagement, communication and support for emotion‐ and self‐regulation skills	Interaction	Verbal de‐escalation and communication	39 (7.7)
		Emotion‐regulation skills and calming techniques	28 (5.5)

**TABLE 3:** Higher‐order categories of workplace violence prevention reported by nurse managers in incident reports (*n* = 524).

Overarching category	Main category	Higher‐order category	Reports, *n* (%)
Patients' clinical stabilisation and load management	Clinical interventions	Optimising medication	108 (20.6)
		Adjusting ECT treatment	22 (4.2)
	Daily load regulation	Stimulus control	41 (7.8)
		Psychoeducation and guidance	5 (1.0)
Resources, competence and workforce management	Staffing and coverage	Nurse staffing levels	44 (8.4)
		Nursing staff work experience	19 (3.6)
		Nursing staff shift planning	18 (3.4)
		Use of security guard services	11 (2.1)
	Maintaining competence	Training and orientation	41 (7.8)
		Nursing staff's knowledge and competence	29 (5.5)
		Additional support	7 (1.3)
Anticipation and standardisation	Dynamic risk assessments and planning	Assessment routines	43 (8.2)
		Task plans	40 (7.6)
		Anticipation of workplace violence	32 (6.1)
	Consistent ward rules	Consistent policies and procedures	25 (4.8)
		Transparency in communication	10 (1.9)
Environment and safety technology	Structures and devices	Doors and access control	72 (13.7)
		Alarms and locations	6 (1.1)
	Protective and restrictive equipment	Personal protective equipment	22 (4.2)
		Ensuring quality and checking restraints	55 (10.5)
		Safe seclusion solutions	57 (10.9)
Patient restrictions and control of external risks	External risk inputs	Substances and parcels	52 (9.9)
		Visits and outsiders	11 (2.1)
	Communication channels and habits	Phone and social media practices	18 (3.4)
		Smoking practices	8 (1.5)
Patient placement and work organisation	Placement solutions	Patient ward transfers	36 (6.9)
		Patient placement on the ward and the layout of patient rooms	19 (3.6)
Information flow, debriefing and organisational learning	Internal communication and support	Reporting and meetings	176 (33.6)
		Debriefings and nursing staff support	16 (3.1)
	Process improvement	Monitoring and feedback	26 (5.0)
		Stakeholder collaboration	28 (5.3)

Through abstraction, we derived a set of conceptual categories describing the preventive measures and prerequisites articulated by nursing staff and nurse managers on forensic psychiatric wards. The categories emerged from the data; no a priori theoretical framework was applied.

To support transparency, we also performed a descriptive quantification of the qualitative categories (Tables 2 and 3). After the inductive content analysis, we counted how many incident reports contained at least one meaning unit coded into each higher‐order category and reported these as *n* (%). The counts therefore represent the proportion of reports in which a theme was documented, not the proportion of all meaning units. Because a single report could include multiple meaning units and could be coded to several categories, the percentages do not sum to 100%. This quantification was used to indicate how commonly categories appeared in the data and does not imply that more frequent categories are necessarily more important.

## 4 Results

### 4.1 Overview of the WPV Prevention Content in Incident Reports

Across the 5‐year study period, the WPV prevention‐oriented free‐text fields provided by nursing staff (*n* = 509) and nurse managers (*n* = 524) described WPV prevention as an interconnected entity covering clinical, relational, procedural and environmental domains.

**FIGURE 2:**
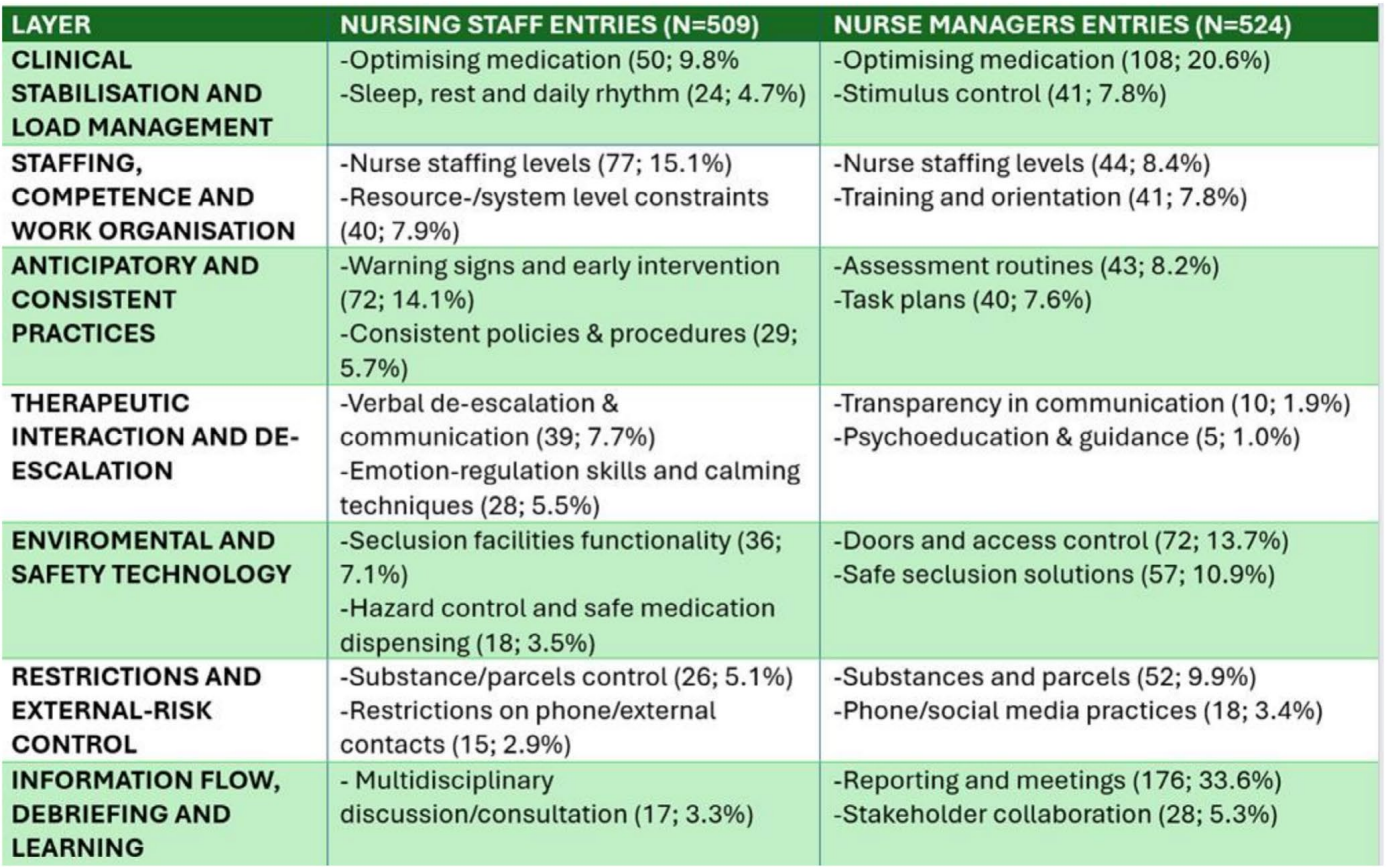
Layered prevention of workplace violence on forensic psychiatric wards: Most frequent higher‐order categories by reporter group.

Figure 2 summarises the layered WPV prevention model derived from the incident report narratives and, where applicable, highlights the most frequently occurring higher‐order categories within each layer for nursing staff and nurse managers' entries. Tables 2 and 3 present the full set of higher‐order categories identified in nursing staff and nurse managers' incident report entries including the number and proportion of reports in which each category appeared.

Overall patterns differed by reporter role: nursing staff entries most often emphasised WPV prevention embedded in everyday care (e.g., nurse staffing levels, recognition of warning signs and early intervention, and verbal de‐escalation and communication), whereas nurse managers' entries more frequently described system‐level WPV prevention (e.g., reporting and meetings, doors and access control, and post‐incident safety measures such as safe seclusion solutions and restraint quality checks).

### 4.2 Clinical Stabilisation and Sleep, Rest and Daily Rhythm

Both groups linked workplace violence prevention to stabilising patients' mental state and reducing stressors. In nursing staff entries, the most frequent clinical category was optimising medication (50; 9.8%), often referring to medication review and intensification, followed by adjusting ECT treatment (23; 4.5%) and sleep, rest and daily rhythm (24; 4.7%), reflecting attention to strain regulation and daily rhythm.

Nursing staff entries also noted that when the patient's mental state was markedly unstable, WPV prevention could be difficult despite staff efforts. Nurse managers described clinical stabilisation even more prominently, most frequently documenting optimising medication (108; 20.6%) and stimulus control (41; 7.8%). Manager reports often framed WPV prevention as a coordinated, multidisciplinary process involving ward teams and physicians, sometimes explicitly linking symptom fluctuation, daily rhythm and escalating risk.

Nurse 1 ‘When the patient's mental state is this poor, it is very difficult to prevent these situations’.

Nurse 2 ‘The patient's refusal to take medication exacerbates their psychotic symptoms, so the patient needs to receive appropriate medication’.

Manager 1 ‘We discuss this in ward rounds and reports; the patient's medication needs urgent review’.

Manager 2 ‘Discussed in relation to this report: recently the patient has been getting “stuck” more often in different situations, and in these moments the risk of aggression is recognised as higher. ECT treatment? Daily rhythm? Is the patient overly tired in the evenings? Rest periods during the day?’

### 4.3 Staffing, Competence and Work Organisation

Staffing was a prominent theme in nursing staff entries describing WPV prevention. The most frequent higher‐order category was *Nurse staffing levels* (77; 15.1%), followed by *resource‐ and system‐level constraints* (40; 7.9%) and *nursing staff work experience* (30; 5.9%).

In nursing staff entries, evenings, nights and high‐risk care situations such as seclusion and transfers were particularly emphasised, and some accounts highlighted the protective value of having personnel familiar with the patient and unit routines. References to the presence of male nurses were infrequent (19; 3.7%).

Nurse managers also described staffing‐related responses, although less frequently than nursing staff including nurse staffing levels (44; 8.4%), shift planning (18; 3.4%) and training and orientation (41; 7.8%). In addition, managers more often documented organisational decisions related to patient placement and ward transfers (e.g., patient ward transfers 36; 6.9%; patient placement on the ward and the layout of patient rooms 19; 3.6%), reflecting responsibility for risk management at system level.

Nurse 3 ‘A sufficient number of nursing staff are present and available when the patient is taken out of restraints, for example for washing or for supervised walks in the courtyard’.

Nurse 4 ‘When planning shift staffing levels, it would be important to ensure that each shift includes enough “regular” experienced staff relative to temporary or substitute staff’.

Manager 3 ‘A new patient… immediate 24h observation and placement in a safer room; we considered transfer to another ward due to space and risk’.

Manager 4 ‘It was agreed at the beginning of the week that the ward's night staffing would be temporarily increased by one additional nurse as a precaution against violent incidents’.

### 4.4 Anticipatory and Consistent/Standardised Practices for High‐Risk Situations

Anticipation and standardisation formed another core domain of WPV prevention. In nursing staff entries, the most frequent anticipatory element was recognition of warning signs and early intervention (72; 14.1%). Preventive work was also described through consistent policies and procedures (29; 5.7%) and practical arrangements that supported timely response in escalating situations such as work allocation and one‐to‐one observation arrangements (24; 4.7%).

Nurse managers also emphasised structured practices including assessment routines (43; 8.2%), task plans (40; 7.6%) and clear and consistent policies and procedures (25; 4.8%). Their reports commonly documented that preventive routines were reviewed and reinforced after incidents, sometimes through training reminders and staff briefings.

Nurse 5 ‘A work plan should be known in advance by everyone, for example the patient undresses in their room and their hands are secured to the shower chair immediately, so the patient's hands are free for less time. In the situation, the sequence of tasks is clear to everyone and there is no need to stop and think about what would be sensible, as that could increase the patient's uncertainty’.

Nurse 6 ‘Paying attention to and recognising the patient's warning signs is key. However, a violent incident is not always predictable’.

Manager 5 ‘Unified procedures are reviewed in violence management training’.

Manager 6 ‘We will continue primary nurse–patient discussions and implement the patient's care according to the plan. It is important to assess what is increasing the patient's distress and why the patient attempts to resolve issues by harming staff, and what type of violence‐related background is involved’.

### 4.5 Therapeutic Interaction and De‐Escalation

In nursing staff entries, WPV prevention was described through verbal de‐escalation and communication (39; 7.7%) and emotion‐regulation skills and calming techniques (28; 5.5%). Nursing staff portrayed WPV prevention as relational and skills‐based, focusing on in‐the‐moment responses and supporting patients' coping and self‐management skills in everyday ward life.

In nurse managers' entries, interaction‐focused strategies were less frequent and were typically described within broader accounts of post‐incident actions and follow‐up. Where explicitly documented, they most commonly referred to Transparency in communication (10; 1.9%) and Psychoeducation and guidance (5; 1.0%). System‐level processes such as reporting and meetings and post‐incident debriefings are described in more detail in Section 4.7 (Information flow, debriefing and organisational learning).

Nurse 7 ‘We aim to identify triggers for violent behaviour and ways to anticipate situations (recognition and finding alternative actions)’.

Manager 7 ‘A new guideline was communicated to patients in a ward meeting and shared with staff in a personnel meeting’.

Manager 8 ‘The treatment approach was changed toward more positive reinforcement and activity‐based interventions’.

### 4.6 Environmental and Safety Technology, Restrictions and External‐Risk Control

Both groups described environmental and restriction‐related measures, but in nurse managers' entries these were documented more frequently and more concretely as implemented responses. Nursing staff mentioned safe seclusion and functionality of seclusion facilities (36; 7.1%), restraints and restraint practices (15; 2.9%) and practical risk controls such as control of substances and restrictions on incoming parcels (26; 5.1%).

Nurse managers frequently described physical and technological safety measures such as doors and access control (72; 13.7%), safe seclusion solutions (57; 10.9%) and substances and parcels (52; 9.9%). These entries often included explicit documentation of what was discussed, what was changed, and how practices would be reinforced.

Nurse 8 ‘The introduction of substances onto the wards should be prevented. Restrictive measures may be necessary for patients whose behaviour is clearly evident and recurrent. Patients with severe substance‐use problems should be placed in a structured ward. This way, they would not hinder the treatment and rehabilitation of patients who might not otherwise become involved with substances in a hospital setting where substances would not be available or tempting. Efforts should focus on preventing substances from reaching the wards, for example by installing motion‐sensor lights in the walking yards’.

Manager 9 ‘The patient was cared for over the weekend through the seclusion room hatch and was transferred at the beginning of the week to another ward with a more modern seclusion facility, where the patient can be managed more safely’.

### 4.7 Information Flow, Debriefing and Organisational Learning

In nursing staff entries, information flow and organisational learning did not form a distinct overarching category; related content was limited and typically embedded within resources and work organisation. A small number of entries referred to multidisciplinary discussion/consultation (17; 3.3%), usually to coordinate roles and agree next steps.

In nurse managers' entries, information flow, debriefing and organisational learning were most often documented through reporting and meetings (176; 33.6%). Managers also described follow‐up practices aimed at supporting staff and reinforcing preventive practices including stakeholder collaboration (28; 5.3%), monitoring and feedback (26; 5.0%) and debriefings and nursing staff support (16; 3.1%). Entries commonly recorded agreed follow‐up actions and how preventive practices would be reinforced.

Overall, nurse managers' entries indicated that incidents were not only recorded but also discussed in meetings, debriefings and shift reports, and in some cases linked to specific follow‐up actions. However, the reports did not enable assessment of the subsequent implementation or effectiveness of these actions.

Nurse 9 ‘It would be important to hold a multidisciplinary discussion on how the patient can be managed safely going forward and which practices need to be changed’.

Manager 10 ‘The incident was processed in a debriefing… we concluded that information flow did not work… and agreed concrete changes for future outings.’

Manager 11 ‘The matter will be discussed during medical rounds and in shift reports. This is a long‐term patient who has previously shown similar difficulties with understanding. Poor insight into their illness further aggravates the situation’.

## 5 Discussion

This study identified workplace violence prevention‐related content in incident report entries by nursing staff and nurse managers in a Finnish forensic psychiatric hospital. Across both groups, WPV prevention was described as a layered entity covering clinical stabilisation, staffing and competence, anticipatory practices, therapeutic interaction, environmental and safety measures, restrictions and external‐risk control, and post‐incident information flow, debriefing, and follow‐up actions. Role‐related differences were also evident: nursing staff emphasised everyday situational WPV prevention, whereas nurse managers more often documented reporting and meetings, debriefings, and follow‐up actions after incidents.

Although incident reports are completed after an event, the WPV prevention‐oriented narrative fields provided a useful qualitative source for examining how staff described WPV prevention in relation to recurrence. These entries may also help to make visible the practice‐based tacit knowledge embedded in everyday forensic ward practice, particularly through descriptions of modifiable antecedents, immediate preventive responses and planned follow‐up actions. The layered character of WPV prevention was derived inductively from the incident report narratives and indicates that preventive domains were described as interrelated rather than separate.

In this study, WPV prevention was frequently linked to active stabilisation of the patient's mental state including medication review and other treatment adjustments. This is consistent with earlier work indicating that violence risk is associated with clinical instability and contextual stressors (Bhavsar et al. 2020; Moulin et al. 2019; Wolf et al. 2023). Nursing staff entries also reflected earlier observations that staff often attribute WPV incidents to acute mental disorder and symptom fluctuation (Dickens et al. 2013; Pulsford et al. 2013). At the same time, the incident report data add practice‐level specificity by showing how these clinical considerations were translated into concrete ward actions and follow‐up decisions including attention to strain, sleep, rest and daily rhythm.

In nursing staff entries, nurse staffing levels emerged as a prominent WPV prevention category, particularly in relation to evenings, nights and high‐risk situations such as seclusion and transfers, underscoring the importance of adequate staffing and allocation. Rather than being framed solely as a resource issue, nurse staffing levels were linked in the incident report entries to immediate preventive capacity, particularly the ability to recognise warning signs early and to maintain consistent approaches during escalating situations. These descriptions correspond with prior findings that low staffing levels and limited experience are associated with increased WPV risk (Sato et al. 2017; Yang et al. 2018; Fute et al. 2015; Schlup et al. 2021), and with evidence linking staff strain, burnout and overtime to vulnerability (Weltens et al. 2021). In this material, resource‐ and system‐level constraints were repeatedly described by staff as factors that may contribute to escalation and staff fatigue, reinforcing the importance of staffing decisions for occupational safety and for the feasibility of WPV prevention in practice.

A small minority of nursing staff reports noted male nurses as supportive in high‐risk situations; however, evidence is mixed, as male nurses may also face higher WPV exposure due to role allocation in violent incidents (Weltens et al. 2021; Doedens et al. 2022; Yosep et al. 2023). Given its limited prominence here, this should be interpreted cautiously as a contextual observation rather than a generalisable WPV prevention strategy.

Anticipation and standardisation formed another core WPV prevention domain. Across incident report entries completed by nursing staff and nurse managers, early identification of warning signs, consistent ward rules, and agreed routines for predictable high‐risk moments were described. This aligns with evidence that systematic risk assessment and structured documentation support anticipation and organisational learning (Cowman et al. 2017; Ogonah et al. 2023), and with evidence from forensic inpatient care suggesting that implementing a structured, ward‐wide model can support ward climate and safety, and may reduce conflict events (Maguire et al. 2018).

Environmental and technological measures were also documented as WPV prevention, and in nurse managers' entries these were often described as concrete implemented measures following incidents. This is consistent with previous research linking ward design and security technologies with WPV management (Nijman et al. 2011; van der Schaaf et al. 2013; Ireland et al. 2019).

Therapeutic interaction was also documented as a WPV prevention layer, particularly in nursing staff descriptions of de‐escalation, consistent communication, and supporting patients' self‐regulation. This is consistent with evidence that interaction skills and knowledge of the patient have preventive value (Yang et al. 2018; Weltens et al. 2021, 2023) and with relational safety perspectives in inpatient mental health settings (Pulsford et al. 2013; Lantta et al. 2016; Chester et al. 2017). Notably, in this dataset, interactional WPV prevention did not displace structural measures such as staffing and procedures; rather, it appeared as a complementary component that staff considered necessary for WPV prevention to work in practice.

Nurse managers' entries in reports, alongside interactional strategies, placed additional emphasis on restrictions and external‐risk management. Restrictions were documented as pragmatic tools for risk control (e.g., managing substances, parcels, visits and movement), but the broader narrative across the reports suggests a tension that is well recognised in the literature: Restrictive practices may reduce immediate risk, yet poorly communicated or inconsistently applied limit setting may increase the likelihood of aggressive responses, whereas empathic and authoritative limit setting is associated with reduced aggression (Maguire et al. 2014). Restrictions on forensic psychiatric patients should be used only as a last resort, be necessary and proportionate, and last no longer than required (O'Donovan and Carballedo 2023). However, seclusion may be necessary, particularly in situations involving substance use or attempts to smuggle substances, as substance use increases forensic psychiatric patients' vulnerability to violent behaviour and, according to Finnish research, to mortality (Ojansuu et al. 2019, 2023).

The most distinctive finding concerns information flow, debriefing and organisational learning documented by nurse managers. In this material, incident report entries suggested that the reporting system served not only as a register of adverse events but also as a platform for articulating WPV prevention knowledge and prompting practice‐oriented follow‐up, as reflected in descriptions of meetings, shift reports, debriefings and subsequent actions. This is consistent with prior observations that reporting culture and feedback support learning and staff safety (Kuosmanen et al. 2019, 2022; Mishina et al. 2023; Russotto et al. 2024). However, the present findings add a practical detail: nurse managers' entries often documented planned or reported follow‐up actions after incidents such as staffing adjustments, changes in patient placement, debriefings and revised communication practices. These entries suggest that incident reports may capture how organisations intend to respond to incidents in practice, although the present data do not show how consistently such actions were implemented or whether they reduced later violence.

Based on these findings, reporting systems could be strengthened to better support WPV prevention and organisational learning. In particular, WPV templates could include structured prompts that encourage documentation of antecedents and early warning signs, interactional strategies used, staffing context and task conditions, and environmental contributors, alongside a clearly documented set of implemented measures and follow‐up actions.

Differences between nursing staff and nurse managers' reporting were consistent with their roles: nursing staff tended to document situational and interactional WPV prevention embedded in real‐time care, whereas nurse managers more often described system‐level prevention through information flow, debriefing practices, staffing decisions and documented follow‐up measures. These role‐related differences indicate that incident reports may contribute to organisational learning by capturing both immediate frontline perspectives and post‐incident managerial responses.

At a practical level, the findings suggest that WPV prevention in forensic psychiatric wards is best understood as an integrated, layered entity in which clinical stabilisation of patients, staffing, competence and work organisation, anticipatory and consistent practices, therapeutic interaction and de‐escalation, environment and safety technology, restrictions and external‐risk control and information flow, debriefing and organisational learning support one another. At the organisational level, the findings point to the importance staff placed on adequate staffing and training during high‐risk periods, as well as on reporting practices that support discussion, follow‐up and review after incidents.

## 6 Limitations

The study has several limitations. First, the dataset comes from a single forensic psychiatric hospital, which limits transferability because organisational culture and facility design may differ across hospitals and countries. Second, independently filed WPV incident reports may lead to underreporting, and the focus and manner of documentation can vary by reporter. Third, only reports from nursing staff and nurse managers were included, so reports from other professional groups are absent from the dataset. Although the incident reporting system includes structured descriptors and contextual details of each event (e.g., unit, time, location and incident circumstances), the present analysis focused on the WPV prevention‐oriented narrative fields and the implemented‐measures sections. In addition, although nurse managers' entries documented follow‐up measures, the dataset did not allow us to assess whether these measures were implemented as planned or whether they affected subsequent violence. As these free‐text fields did not consistently capture detailed antecedents, the incident‐level forensic detail reported in this study was limited. In addition, one member of the research team was employed by the study hospital; while this may have influenced interpretations, familiarity with the forensic psychiatric context also supported a nuanced understanding of the data.

## 7 Conclusions

This study suggests that WPV prevention‐oriented narrative fields in incident reports may help make visible practice‐based, tacit knowledge reflected in how nursing staff and nurse managers describe WPV prevention in forensic psychiatric inpatient care. In this Finnish forensic hospital, WPV prevention was described as a layered entity involving clinical stabilisation, staffing and competence, anticipatory practices, therapeutic interaction, environmental and safety measures, restrictions and external‐risk control, and post‐incident information flow, debriefing, and follow‐up actions. Nurse managers' entries particularly emphasised reporting and meetings, debriefings and follow‐up actions after incidents. Further research is needed to examine how documented actions are implemented in practice and whether they are associated with subsequent reductions in violence.

## Ethics Statement

The reporting dataset contained no patient names and no direct personal identifiers. The dataset contained occasional staff names, which were removed prior to analysis. After this, the researchers had access only to anonymised, exported incident report data. The dataset then contained no direct identifiers, and the analysis focused on narrative text content rather than individual cases. A privacy notice and a data protection impact assessment were prepared for the study. Under Finnish legislation, a separate ethical review was not required (Finnish National Board on Research Integrity, TENK 2019). The data were obtained after applying for a research permit from the hospital, which was granted by the hospital's chief physician. The security manager provided the data to the researcher.

## Conflicts of Interest

The authors declare no conflicts of interest.

## Data Availability

The authors have nothing to report.
